# Comparing ChatGPT and DeepSeek for Assessment of Multiple-Choice Questions in Orthopedic Medical Education: Cross-Sectional Study

**DOI:** 10.2196/75607

**Published:** 2025-12-19

**Authors:** Chirathit Anusitviwat, Sitthiphong Suwannaphisit, Jongdee Bvonpanttarananon, Boonsin Tangtrakulwanich

**Affiliations:** 1 Department of Orthopedics, Faculty of Medicine, Prince of Songkla University, 15 Karnchanavanich Road, Hat Yai, 90110, Thailand, 66 74451601; 2 Department of Orthopaedics, Faculty of Medicine, Vajira Hospital, Navamindradhiraj University, Bangkok, Thailand

**Keywords:** ChatGPT, large language model, LLM, orthopedic, multiple-choice question, MCQ

## Abstract

**Background:**

Multiple-choice questions (MCQs) are essential in medical education for assessing knowledge and clinical reasoning. Traditional MCQ development involves expert reviews and revisions, which can be time-consuming and subject to bias. Large language models (LLMs) have emerged as potential tools for evaluating MCQ accuracy and efficiency. However, direct comparisons of these models in orthopedic MCQ assessments are limited.

**Objective:**

This study compared the performance of ChatGPT and DeepSeek in terms of correctness, response time, and reliability when answering MCQs from an orthopedic examination for medical students.

**Methods:**

This cross-sectional study included 209 orthopedic MCQs from summative assessments during the 2023‐2024 academic year. ChatGPT (including the “Reason” function) and DeepSeek (including the “DeepThink” function) were used to identify the correct answers. Correctness and response times were recorded and compared using a *χ*^2^ test and Mann-Whitney *U* test where appropriate. The two LLMs’ reliability was assessed using the Cohen κ coefficient. The MCQs incorrectly answered by both models were reviewed by orthopedic faculty to identify ambiguities or content issues.

**Results:**

ChatGPT achieved a correctness rate of 80.38% (168/209), while DeepSeek achieved 74.2% (155/209; *P*=.04). ChatGPT’s Reason function also outperformed DeepSeek’s DeepThink function (177/209, 84.7% vs 168/209, 80.4%; *P*=.12). The average response time for ChatGPT was 10.40 (SD 13.29) seconds, significantly shorter than DeepSeek’s 34.42 (SD 25.48) seconds (*P*<.001). Regarding reliability, ChatGPT demonstrated an almost perfect agreement (κ=0.81), whereas DeepSeek showed substantial agreement (κ=0.78). A completely false response was recorded in 7.7% (16/209) of responses for both models.

**Conclusions:**

ChatGPT outperformed DeepSeek in correctness and response time, demonstrating its efficiency in evaluating orthopedic MCQs. This high reliability suggests its potential for integration into medical assessments. However, our results indicate that some MCQs will require revisions by instructors to improve their clarity. Further studies are needed to evaluate the role of artificial intelligence in other disciplines and to validate other LLMs.

## Introduction

Multiple-choice questions (MCQs) are a cornerstone of medical education, particularly for assessing knowledge acquisition and clinical reasoning in specialized fields such as orthopedic medicine. Traditionally, MCQs have been refined through expert reviews and iterative revisions, a process that can be time-consuming and prone to human bias [1,2]. MCQs are widely used in medical education because of their efficiency, objectivity, and ability to assess a broad range of knowledge in a standardized manner [2,3].

Large language models (LLMs), such as ChatGPT and DeepSeek, have emerged as potential tools for evaluating MCQ accuracy and efficiency. These artificial intelligence (AI)–driven approaches can systematically assess question clarity, difficulty, and alignment with learning objectives [4]. However, research comparing the performance of different LLMs in answering orthopedic MCQs remains limited.

Recent studies have highlighted the capabilities of LLMs in medical education [5-7]. These studies explored the use of GPT-4 for formative and summative assessments in medicine, demonstrating its potential in evaluating medical knowledge and providing feedback to students. Additionally, ChatGPT has shown notable accuracy in medical education, particularly in answering MCQs for licensing exams [8]. Prior studies have shown its ability to perform well in both basic and clinical sciences, with accuracy rates ranging from 70% to 74% [9]. Despite its potential, the reliability of LLMs in MCQ assessment, particularly in disciplines requiring complex clinical reasoning, remains an area of ongoing investigation [8,9].

DeepSeek V3, a recent advancement in LLMs, has shown promise in various domains in health care. Research has explored its use in the medical context, including patient education on asthma, ocular oncology, and laparoscopic cholecystectomy [10-12]. However, no study has evaluated the performance of DeepSeek V3 as an assessment tool for the quality of MCQs in medical education.

This study compared the performance of ChatGPT and DeepSeek in answering orthopedic MCQs by evaluating their correctness, response time, and reliability. These findings provide insights into the feasibility of integrating LLMs into medical student assessments and highlight areas where MCQs may require refinement.

## Methods

### Study Design

This cross-sectional study was conducted at the Department of Orthopedics, Faculty of Medicine, Prince of Songkla University, Thailand. It focused on MCQs used in the summative assessment of orthopedic rotations during the 2023‐2024 academic year. All MCQs from the question pool during this period were included. As this was an educational study, there were no criteria for participant withdrawal or study termination. Instead, all available MCQs from the study period were analyzed.

### Assessment Tools

In this study, MCQs were defined as structured assessment tools consisting of a question stem and multiple answer choices with one correct response. This study evaluated the ability of two LLMs, ChatGPT and DeepSeek, to analyze and process these questions. The versions used were GPT-4o for ChatGPT and and DeepSeek-V3 for DeepSeek, as both represented the most stable and publicly accessible variants during the data collection period. The two LLMs were accessed between January 2025 and March 2025.

GPT-4o was developed by OpenAI and released in May 2024 [13]. This model was optimized for faster response times and improved performance while maintaining high accuracy in complex reasoning tasks. Additionally, ChatGPT includes a “Reason” function that allows users to request explanations for its responses, providing insights into the logic behind its choices. This feature enhances transparency and facilitates a deeper understanding of how the model arrived at its conclusions, making it particularly useful for educational and assessment purposes [14].

DeepSeek-V3, another advanced LLM used in medicine, was initially released in December 2024 [13]. It offers enhanced performance with improved accuracy, faster processing, and a broad, up-to-date knowledge base [15]. It includes a “DeepThink” function designed to enhance logical reasoning by breaking down complex problems step by step. The AI model is designed to deliver high-quality, reliable, and efficient assistance in various applications. It excels in natural language processing, providing superior contextual understanding and multilingual support, ensuring more natural and coherent interactions.

### Data Collection and Evaluation Procedures

This study involved 3 main steps. First, a set of MCQs from orthopedic assessments conducted in 2023‐2024 was collected and reviewed for quality. These MCQs were created by orthopedic faculty members and approved by the orthopedic committee to ensure content alignment with curricular objectives, and they have been used in orthopedic examinations for more than 5 years. Next, each MCQ was input into both ChatGPT and DeepSeek. The AI responses, along with the accuracy and consistency data, were recorded. The Reason and DeepThink functions were tested, and response times were documented. Finally, any MCQs for which both LLMs failed to identify the correct answer were reviewed by a panel of orthopedic faculty members. These questions were refined to improve clarity and adjusted to ensure an appropriate level of difficulty. To determine reliability, all the MCQs and their answer keys were input into both ChatGPT and DeepSeek by two orthopedic faculty members, who both had experience in medical education at an academic hospital, on separate days.

### Data Analysis

Statistical methods were used to compare the performance of the two LLMs. Correctness rates were expressed as percentages and compared using the *χ*^2^ test. Processing times were reported as means with SDs and analyzed using either a 2-sample, 2-tailed *t* test or a Mann-Whitney *U* test, depending on data distribution. The interrater reliability between the two LLMs was evaluated using the Cohen κ coefficient. Statistical significance was set at *P*<.05.

### Ethical Considerations

This study was conducted in accordance with the ethical principles of the Declaration of Helsinki, and the protocol was reviewed and approved by the Institutional Review Board of the Faculty of Medicine at Prince of Songkla University (REC. 68-097-11-1). This study involved only secondary analysis of existing MCQs and contained no participant identifiers; thus, individual informed consent was waived. As this study did not involve direct participation by human subjects, no compensation was provided. Finally, no images or materials that could identify individual participants were included in the manuscript or supplementary files.

## Results

A total of 209 orthopedic MCQs were analyzed across 11 subspecialty categories to compare the accuracy of ChatGPT and DeepSeek (Table 1) and the Reason and DeepThink functions (Table 2). Overall, ChatGPT achieved a significantly higher total accuracy of 80.4% (168/209) compared with 74.2% (155/209) for DeepSeek (*P*=.04). Although no single subspecialty demonstrated a significant difference after stratification, ChatGPT showed numerically superior accuracy across most categories, with the largest gaps observed in the tumor and infection (92.9% vs 71.4%) and lower-limb injury (68.8% vs 50.0%) domains. These findings indicate that ChatGPT outperformed DeepSeek in overall accuracy for orthopedic MCQs.

In the second comparison, the Reason function in ChatGPT demonstrated an accuracy of 84.7% (177/209), while the DeepThink function in DeepSeek achieved 80.4% (168/209) accuracy. However, the difference was not statistically significant (*P*=.12). Nevertheless, the Reason function seems to have performed better in some domains, such as the pediatric and anomalous category (80.0% vs 65.0%) and the tumor and infection category (92.9% vs 78.6%), whereas DeepThink showed slightly higher accuracy in the complications in orthopedics category (90.9% vs 63.6%). However, these discrepancies did not appear to correlate with any specific subspecialty or content category.

**Table 1.** Comparison of accuracy between ChatGPT and DeepSeek across orthopedic multiple choice question (MCQ) categories (n=209).

MCQs in each category	ChatGPT responses, n (%)	DeepSeek responses, n (%)	*P* value
Upper limb injury (n=28)	23 (82.1)	21 (75.0)	.48
Upper limb disease (n=21)	21 (100.0)	20 (95.2)	.32
Pelvic and spine injury (n=19)	15 (79.0)	14 (73.7)	.56
Lower limb injury (n=16)	11 (68.8)	8 (50.0)	.18
Lower limb disease (n=12)	11 (91.7)	10 (83.3)	.32
Back and neck problems (n=26)	21 (80.8)	19 (73.1)	.41
Sprain and strain (n=8)	6 (75.0)	7 (87.5)	.32
Pediatric and anomalous (n=20)	12 (60.0)	12 (60.0)	>.99
Tumor and infection (n=14)	13 (92.9)	10 (71.4)	.08
Complications in orthopedics (n=11)	7 (63.6)	9 (81.8)	.16
Rehabilitation and physical therapy (n=34)	28 (82.4)	25 (73.5)	.26
Total (n=209)	168 (80.4)	155 (74.2)	.04

**Table 2.** Comparison of accuracy between “Reason” (ChatGPT) and “DeepThink” (DeepSpeek) functions across orthopedic multiple choice question (MCQ) categories (n=209).

MCQs in each category	Reason responses, n (%)	DeepThink responses, n (%)	*P* value
Upper limb injury (n=28)	25 (89.3)	25 (89.3)	>.99
Upper limb disease (n=21)	21 (100.0)	20 (95.2)	.32
Pelvic and spine injury (n=19)	16 (84.2)	14 (73.7)	.16
Lower limb injury (n=16)	11 (68.8)	9 (56.3)	.41
Lower limb disease (n=12)	11 (91.7)	10 (83.3)	.31
Back and neck problems (n=26)	22 (84.6)	22 (84.6)	>.99
Sprain and strain (n=8)	7 (87.5)	7 (87.5)	>.99
Pediatric and anomalous (n=20)	16 (80.0)	13 (65.0)	.18
Tumor and infection (n=14)	13 (92.9)	11 (78.6)	.16
Complications in orthopedics (n=11)	7 (63.6)	10 (90.9)	.08
Rehabilitation and physical therapy (n=34)	28 (82.4)	27 (79.4)	.56
Total (n=209)	177 (84.7)	168 (80.4)	.12

The most incorrect responses for both ChatGPT and DeepSeek occurred in the complications (2/11, 18.2%), pediatric and anomalous (3/20, 15.0%), and pelvic and spine injury (3/19, 15.8%) categories, reflecting areas where clinical reasoning and management knowledge were more heavily tested. The two lowest rates of mistakes were observed in the tumor and infection and upper limb injury categories. The overall rate of completely false responses from the two LLMs was 7.7% (16/209). Examples of questions that resulted in false responses from both LLMs are provided in Multimedia Appendix 1.

ChatGPT demonstrated a significantly faster response time, with an average processing time of 10.40 (SD 13.29) seconds, compared to DeepSeek’s 34.42 (SD 25.48) seconds (*P*<.001).

Finally, the interrater reliability for the two LLMs was high. ChatGPT achieved a Cohen κ value of 0.81, indicating good agreement, whereas DeepSeek showed substantial agreement, with a κ of 0.78. These findings underscore both the consistency of the LLM-generated responses and a residual variability that warrants continued human oversight in educational and assessment settings.

## Discussion

### Principal Results

This study compared the performance of two advanced LLMs, ChatGPT and DeepSeek, in the processing of orthopedic MCQs regarding their correctness, response time, and reliability. Our findings indicate that ChatGPT outperformed DeepSeek in terms of both accuracy and response time. Additionally, the Reason function of ChatGPT, which provides explanations for selected answers, appears to contribute to its superior performance. Both ChatGPT and DeepSeek exhibited high interrater reliability when generating responses to orthopedic MCQs.

### Comparison With Previous Studies

The results of this study are consistent with a prior study showing that advanced LLMs demonstrated a high level of accuracy in answering orthopedics-related MCQs [16]. In our study, the accuracy of ChatGPT without the Reason function was 80.4%, while a previous study reported an accuracy rate of 70.60%. When using the Reason function in ChatGPT, the accuracy increased to 84.7%. This may imply that the Reason function can promote deeper comprehension and facilitate critical thinking in the medical education setting.

The high accuracy observed for ChatGPT and DeepSeek is particularly valuable in high-stakes environments like medical education [17]. Efficient processing can facilitate the development of high-quality, comprehensive MCQs, minimizing ambiguities in both questions and answer choices to enhance assessment reliability. Furthermore, the ability of ChatGPT’s Reason function to provide detailed explanations adds an educational dimension often missing in traditional assessment tools, thereby facilitating a deeper understanding of complex clinical contexts that mirror real-world practice [18].

The balance between AI efficiency and educational values poses an issue. Although ChatGPT’s speed and accuracy are advantageous, reliance on automated systems for assessment must be balanced with human oversight to ensure that the nuances of clinical reasoning and decision-making are adequately taken into account. This is particularly important in fields such as orthopedic surgery, where clinical decisions can have significant implications for patient care [19].

We found that 7.7% of the MCQs yielded false responses from both LLMs, warranting further examination. This finding suggests that there may be inherent issues in question construction, leading to ambiguity and misinterpretation. Poorly constructed questions can stem from several factors, including unclear wording, imprecise phrasing of the question stem, or answer choices that do not effectively discriminate between correct and incorrect responses [20,21]. By revising ambiguous or poorly constructed questions, educators can improve the overall reliability and validity of assessments, subsequently enhancing the fairness of the evaluation process [22].

Both ChatGPT and DeepSeek exhibited high interrater reliability when generating responses to orthopedic MCQs. These results suggest that both AI models can consistently answer the standardized questions, although some variability remains. The differences in the κ values may have resulted from variations in the training data and model algorithms. Despite their strong reliability, the models did not achieve complete consistency, highlighting the importance of human oversight.

Although AI tools provide notable benefits in terms of efficiency and consistency, there are critical risks associated with overreliance on them for medical education and assessment. Therefore, human supervision is necessary to guarantee that AI functions as a helpful tool rather than a substitute for professional judgment, particularly in situations involving high stakes in education or health care. Educators should carefully combine AI with conventional teaching techniques and evaluation procedures to maintain academic integrity and guarantee that students keep refining their decision-making abilities.

### Limitations

Despite the promising findings, this study had some limitations. Our study focused solely on orthopedic MCQs, which may limit the generalizability of our results to other areas of medical education. Future studies should extend these analyses to include diverse medical disciplines to better understand the broader applicability of AI-assisted assessments. Additional limitations include the study’s cross-sectional design, which only captured the LLMs’ performance at a single point in time. Given that LLMs such as ChatGPT and DeepSeek are continually updated and improved, their performance may vary over time. Furthermore, while the LLMs provided explanations for selected answers, we did not formally assess the quality of these explanations. Implementing a structured evaluation of explanation quality in future studies could offer valuable educational perspectives.

### Implications of the Findings

The findings suggest that AI tools like ChatGPT and DeepSeek can enhance the efficiency of medical assessments in orthopedic rotation. ChatGPT demonstrated higher accuracy and faster responses compared to DeepSeek. Furthermore, both LLMs could help identify poorly constructed MCQs. However, reevaluation by teachers or assessors remains essential to ensure that responses are clinically relevant and that results of summative assessments are accurate.

For broader application in other medical specialties, we propose the following practical steps. First, validated, specialty-specific MCQs should be gathered from the target discipline to ensure content relevance and quality. Second, these questions should be input into selected AI models and their responses systematically recorded and evaluated for accuracy. Third, to ensure reliability, at least two faculty experts should independently assess the LLM-generated answers, with any discrepancies resolved through consensus review. Fourth, poorly performing or ambiguous MCQs should be identified and refined for clarity.

Currently, there are a large number of advanced LLMs. Future studies should systematically compare other AI tools to determine which models perform best in MCQ assessment. Additionally, future research should be conducted to explore whether AI assistance could be used for other types of assessments, including modified essay questions, objective structured clinical examinations, or key-feature questions.

### Conclusions

In the study, ChatGPT outperformed DeepSeek in answering orthopedic MCQs in terms of overall accuracy and response time, although both models exhibited strong interrater reliability. Specifically, ChatGPT achieved a significantly higher accuracy rate compared with DeepSeek, and its Reason function further enhanced performance. These findings suggest that ChatGPT can serve as a valuable tool in medical education and assessment, particularly when using explanatory reasoning capabilities.

Despite these encouraging results, both AI models showed areas of weakness, particularly in complex domains such as complications, pediatrics and anomalies, and pelvic and spine injuries. These errors highlight the importance of maintaining human oversight when using LLMs for educational or evaluative purposes. Thus, the integration of AI tools should be viewed as complementary to, rather than a replacement for, expert clinical judgment and expertise.

## Supplementary material

10.2196/75607 Multimedia Appendix 1 Examples of questions that resulted in false responses from both ChatGPT and DeepSeek.

## Abbreviations

AI: artificial intelligence
LLM: large language model
MCQ: multiple-choice question

## References

[1] Al-Rukban MO (2006). Guidelines for the construction of multiple choice questions tests. J Family Community Med.

[2] Olayemi E (2013). Multiple choice questiones as a tool for assessment in medical education. Ann Biomed Sci.

[3] Parekh P, Bahadoor V (2024). The utility of multiple-choice assessment in current medical education: a critical review. Cureus.

[4] Kayaalp ME, Prill R, Sezgin EA, Cong T, Królikowska A, Hirschmann MT (2025). DeepSeek versus ChatGPT: multimodal artificial intelligence revolutionizing scientific discovery. From language editing to autonomous content generation-redefining innovation in research and practice. Knee Surg Sports Traumatol Arthrosc.

[5] Mehta S, Haddad EN, Burke IB (2025). Assessment of large language model performance on medical school essay-style concept appraisal questions: exploratory study. JMIR Med Educ.

[6] Krumsvik RJ (2025). GPT-4’s capabilities for formative and summative assessments in Norwegian medicine exams-an intrinsic case study in the early phase of intervention. Front Med (Lausanne).

[7] Kondo T, Nishigori H (2025). AI’s accuracy in extracting learning experiences from clinical practice logs: observational study. JMIR Med Educ.

[8] Gonsalves C (2023). On ChatGPT: what promise remains for multiple choice assessment?. J Learn Dev High Educ.

[9] Meo SA, Al-Masri AA, Alotaibi M, Meo MZS, Meo MOS (2023). ChatGPT knowledge evaluation in basic and clinical medical sciences: multiple choice question examination-based performance. Healthcare (Basel).

[10] Liu Y, Yu F, Zhang X (2025). Assessing the role of large language models between ChatGPT and DeepSeek in asthma education for bilingual individuals: comparative study. JMIR Med Inform.

[11] Das D, Narayan A, Mishra V (2025). AI chatbots in answering questions related to ocular oncology: a comparative study between DeepSeek v3, ChatGPT-4o, and Gemini 2.0. Cureus.

[12] Dincer HA, Dogu D (2025). Evaluating artificial intelligence in patient education: DeepSeek-V3 versus ChatGPT-4o in answering common questions on laparoscopic cholecystectomy. ANZ J Surg.

[13] Xiong L, Wang H, Chen X (2025). DeepSeek: paradigm shifts and technical evolution in large AI models. IEEE/CAA J Autom Sinica.

[14] Khan RA, Jawaid M, Khan AR, Sajjad M (2023). ChatGPT - Reshaping medical education and clinical management. Pak J Med Sci.

[15] Temsah A, Alhasan K, Altamimi I (2025). DeepSeek in healthcare: revealing opportunities and steering challenges of a new open-source artificial intelligence frontier. Cureus.

[16] Gan W, Ouyang J, Li H (2024). Integrating ChatGPT in orthopedic education for medical undergraduates: randomized controlled trial. J Med Internet Res.

[17] Kung TH, Cheatham M, Medenilla A (2023). Performance of ChatGPT on USMLE: potential for AI-assisted medical education using large language models. PLOS Digit Health.

[18] Al Shuraiqi S, Aal Abdulsalam A, Masters K, Zidoum H, AlZaabi A (2024). Automatic generation of medical case-based multiple-choice questions (MCQs): a review of methodologies, applications, evaluation, and future directions. BDCC.

[19] Crebbin W, Beasley SW, Watters DAK (2013). Clinical decision making: how surgeons do it. ANZ J Surg.

[20] Al-Faris EA, Alorainy IA, Abdel-Hameed AA, Al-Rukban MO (2010). A practical discussion to avoid common pitfalls when constructing multiple choice questions items. J Family Community Med.

[21] Przymuszała P, Piotrowska K, Lipski D, Marciniak R, Cerbin-Koczorowska M (2020). Guidelines on writing multiple choice questions: a well-received and effective faculty development intervention. Sage Open.

[22] Downing SM (2005). Threats to the validity of clinical teaching assessments: what about rater error?. Med Educ.

