# Correction: Comparing ChatGPT and DeepSeek for Assessment of Multiple-Choice Questions in Orthopedic Medical Education: Cross-Sectional Study

**DOI:** 10.2196/92549

**Published:** 2026-02-26

**Authors:** Chirathit Anusitviwat, Sitthiphong Suwannaphisit, Jongdee Bvonpanttarananon, Boonsin Tangtrakulwanich

**Affiliations:** 1 Department of Orthopedics, Faculty of Medicine, Prince of Songkla University, 15 Karnjanavanich Road, Hat Yai, 90110, Thailand, 66 74451601; 2 Department of Orthopaedics, Faculty of Medicine, Vajira Hospital, Navamindradhiraj University, Bangkok, Thailand

In “Comparing ChatGPT and DeepSeek for Assessment of Multiple-Choice Questions in Orthopedic Medical Education: Cross-Sectional Study” [1], a typographical error has been found.

In Table 2, the accuracy of the “Reason” function, reported as n (%) in the pelvic and spine injury category, was “16 (68.8%).” On re-examination, the correct accuracy for the pelvic and spine injury category using the “Reason” function was 16 out of 19, corresponding to 84.2%. Thus, the value reported in Table 2 has been revised from “16 (68.8)” to “16 (84.2).” The revised version of Table 2 is shown below (with change shown in italics).

**Table 2.** Comparison of accuracy between “Reason” (ChatGPT) and “DeepThink” (DeepSeek) functions across orthopedic multiple choice question (MCQ) categories (n=209).

MCQs in each category	Reason responses, n (%)	DeepThink responses, n (%)	*P* value
Upper limb injury (n=28)	25 (89.3)	25 (89.3)	>.99
Upper limb disease (n=21)	21 (100.0)	20 (95.2)	.32
Pelvic and spine injury (n=19)	*16 (84.2)*	14 (73.7)	.16
Lower limb injury (n=16)	11 (68.8)	9 (56.3)	.41
Lower limb disease (n=12)	11 (91.7)	10 (83.3)	.31
Back and neck problems (n=26)	22 (84.6)	22 (84.6)	>.99
Sprain and strain (n=8)	7 (87.5)	7 (87.5)	>.99
Pediatric and anomaly (n=20)	16 (80.0)	13 (65.0)	.18
Tumor and infection (n=14)	13 (92.9)	11 (78.6)	.16
Complication in orthopedics (n=11)	7 (63.6)	10 (90.9)	.08
Rehabilitation and physical therapy (n=34)	28 (82.4)	27 (79.4)	.56
Total (n=209)	177 (84.7)	168 (80.4)	.12

The correction will appear in the online version of the paper on the JMIR Publications website, together with the publication of this correction notice. Because this was made after submission to PubMed, PubMed Central, and other full-text repositories, the corrected article has also been resubmitted to those repositories.
