# Supplementary material for: Comparing ChatGPT and DeepSeek for Assessment of Multiple-Choice Questions in Orthopedic Medical Education: Cross-Sectional Study
Source: JMIR Form Res. 2025 Dec 19;9:e75607. doi: 10.2196/75607 (PMC12716854; doi:10.2196/75607)
Supplement: Multimedia Appendix 1 [file formative-v9-e75607-s001.docx]

Examples of questions that resulted in false responses from both ChatGPT and DeepSeek

A newborn boy is diagnosed as bilateral talipes equinovarus. As a general practitioner, what is the most appropriate management?

A. Observation

B. Follow-up at 3 months

C. Start manipulation and cast

D. Refer to Orthopaedic surgeon immediately

E. Refer to Orthopaedic surgeon within one month

A 35 year old man has a closed unstable pelvic fracture with hemodynamic instability. What radiographic finding should we expect to see in this patient?

A. Separation of pubic symphysis 0.5 cm

B. Widening of sacroiliac joint 0.5 cm

C. Fracture of superior and inferior pubic ramus

D. Impacted sacral fracture

E. Avulsion fracture of transverse process of L5

A 58 year old woman diagnosed as fracture of distal end of right radius was treated by short arm cast for 3 days. She came with having pain and swelling of all digits. Physical examination revealed swelling of hand distal to the cast. She has no pain on passive stretching test. What is the most appropriate management?

A. Change the cast

B. Rail road the cast

C. Remove cast and observe at ER

D. Intracompartment pressure monitoring

E. Fasciotomy
